# Use of technology in evidence-based programs for child maltreatment and its impact on parent and child outcomes

**DOI:** 10.3389/fdgth.2023.1224582

**Published:** 2023-07-06

**Authors:** Ashwini Tiwari, Manderley Recinos, Jamani Garner, Shannon Self-Brown, Rushan Momin, Sadhana Durbha, Vanessa Emery, Kathryn O’Hara, Elizabeth Perry, Regan Stewart, Christine Wekerle

**Affiliations:** ^1^ Institute of Public and Preventive Health, Augusta University, Augusta, GA, United States; ^2^ Department of Health Policy and Behavioral Sciences, School of Public Health, Georgia State University, Atlanta, GA, United States; ^3^ Department of Psychiatry and Behavioral Sciences, Medical College of Georgia, Augusta University, Augusta, GA, United States; ^4^ Medical University of South Carolina, Charleston, SC, United States; ^5^ Department of Pediatrics, McMaster University, Hamilton, ON, Canada; ^6^ Department of Psychiatry and Behavioral Neurosciences, McMaster University, Hamilton, ON, Canada

**Keywords:** child maltreatment, technology, scoping review, at-risk parents, trauma treatment, child abuse, child trauma

## Abstract

**Introduction:**

Technology has been used in evidence-based child maltreatment (CM) programs for over a decade. Although advancements have been made, the extent of the application of technology in these programs, and its influence on parental and child outcomes, remains unclear within the context of changes that emerged because of the COVID-19 pandemic. This scoping review provides a contextualized overview and summary of the use of technology in evidence-based parenting and child programs serving families impacted by child maltreatment and the effects of technology-enhanced programs on target outcomes.

**Materials and methods:**

Using Arksey and O'Malley's methodological framework, we searched seven databases to identify peer-reviewed and grey literature published in English from 2000 to 2023 on evidence-based programs, according to the California Evidence-Based Clearinghouse (CEBC), that included technological supports for two populations: at-risk parents for child maltreatment prevention, and children and youth 0–18 years exposed to child maltreatment. All study designs were included.

**Results:**

Eight evidence-based parenting programs and one evidence-based child trauma program were identified as using technology across a total of 25 peer-reviewed articles and 2 peer-reviewed abstracts meeting inclusion criteria (*n* = 19 on parent-level programs; *n* = 8 on child-level programs). Four studies were published in the context of COVID-19. Two main uses of technology emerged: (1) remote programmatic delivery (i.e., delivering all or part of the program virtually using technology) and (2) programmatic enhancement (i.e., augmenting program content with technology). Improvements across parenting and child mental health and behavioral outcomes were generally observed.

**Discussion:**

Technology use in evidence-based child maltreatment programs is not new; however, the small sample since the start of the COVID-19 pandemic in this review that met inclusion criteria highlight the dearth of research published on the topic. Findings also suggest the need for the inclusion of implementation outcomes related to adoption and engagement, which could inform equitable dissemination and implementation of these programs. Additional considerations for research and practice are discussed.

## Introduction

Child maltreatment remains a national public health priority in the US with nearly 600,000 cases substantiated in 2021, and approximately 1,820 deaths related to singular or co-occurring incidents of physical abuse, sexual abuse emotional abuse, and neglect (1). More recent evidence suggests global physical abuse and psychological abuse rates of 18% and 39%, respectively, during the first years of the coronavirus pandemic, COVID-19 (2). Youth exposed to child maltreatment are vulnerable to a host of short- and long-term biological and psychosocial adverse outcomes (3–5). At-risk parents, the primary perpetrators of CM (1), may also be victims of trauma, and experience deficits in executive functioning and emotion regulation that amplify negative parenting behaviors (6–10). High-risk families experience several chronic stressors such as part-time employment, economic hardship, trauma history and mental health symptomology (8, 9). COVID-19 placed exceptional demands on high-risk minority families, who were at not only increased risk for contracting the virus, but at disproportionate risk of unemployment and psychosocial distress attributed to increases in financial and social support instabilities (11). Such disruptions to family routine and external resources have played a role in reports of increased burnout among caregivers (12), and the possibility of abuse against children (13–16).

An abundance of evidence exists for the effectiveness of evidence-based programs in addressing adverse outcomes among caregivers as well as child and youth victims. Of note, behaviorally-based child maltreatment prevention programs are founded on the assumption that child maltreatment risk resulting from parenting skill deficits can be improved by providing parents training in a repertoire of skills, using a specific instructional format that includes: (1) education or instruction on target skills, (2) behavioral modeling of target skills, (3) parent practice of skills through role plays and live practice with the child, and (4) feedback to parents (17). Importantly, these skills have been found to be critical to parent behavior change in a meta-analysis of parent training programs by Kaminski and colleagues (18). Research suggests that parenting programs for caregivers with increased risk of child maltreatment perpetration lead to reductions in child maltreatment potential, such as parental stress via self-report and biomarkers (19–21), as well as improvements in parent-child interactions, home safety, child healthcare skills, and other caregiver (e.g., maternal social support, maternal depression, non-violent discipline) and child outcomes (e.g., adaptive functioning, behavioral risk) (22–28).

Similar improvements are seen among youth engaged in evidence-based psychotherapeutic programs for child and youth trauma associated with child maltreatment exposure, which are often based in behavioral and cognitive restructuring frameworks as well as trauma-focused strategies to address the psychological impact of trauma among youth (29, 30). These programs may use multiple techniques over the course of treatment to change thoughts and behaviors of trauma-affected youth and their caregivers, such as psychoeducation and coping exercises (e.g., relaxation via breathing, meditation). For example, in the gold-standard child-sexual abuse intervention, Trauma-Focused Cognitive Behavioral Therapy (TF-CBT), providers implement components defined by the acronym “PRACTICE”: (1) psychoeducation and parenting, (2) relaxation, (3) affective expression and modulation, (4) cognitive coping, trauma narrative processing, (5) *in vivo* exposure, (6) conjoint parent-child sessions, and (7) enhancing safety (31). Such evidence-based programs are associated with notable changes across child behavior and mental health symptomology, including decreases in post-traumatic stress disorder symptomology, abuse-related fears, sexualized behavior, depression, anxiety, shame, and behavior problems among traumatized youth (29, 32–34).

Despite the proven effectiveness across these programs for both parents and children, reaching and retaining families in real-life practice is a substantial challenge in general. However, the availability and delivery of such scientifically supported programs were critical during the height of the pandemic, as the “pandemic paradox” (35) created by social distancing regulations to reduce spread of infection had unintended consequences for families; indeed, caregivers were placed at greater risk of stress in the household and child maltreatment perpetration. In response to national public health guidelines, agencies offering evidence-based practices rapidly adapted in-person delivery to a virtual delivery approach to prevent disruptions in service provision for vulnerable populations (36). In addition, telehealth guidelines were released for aforementioned well-supported behavioral treatment TF-CBT, which provided considerations for both providers and families on equipment, privacy, internet stability, and safety (37). As such, the pandemic heightened awareness of the utility and importance of digital platforms for services.

The field of child maltreatment prevention and treatment has considered technological platforms for program delivery and augmentation for over a decade prior to COVID-19 (the CDC first funded projects to explore such efforts in 2006), though dissemination and implementation of these approaches were very limited in standard practice (for examples, see the Introduction to the Special Issue on Using Technology to Address Child Maltreatment: Prevention, Intervention, and Research (38, 39). However, almost three years following the pandemic onset, it is unclear how the use of technology in practice has evolved to support child maltreatment evidence-based prevention and intervention program access and effectiveness. Because the pandemic necessitated the use of virtual program delivery, it is imperative that we learn from the data and information gathered prior to and during this period that can inform how we should move forward with leveraging telehealth and other technology to improve program access, efficiency, and effectiveness. For instance, preliminary data collected during 2020 suggest that virtual sessions are feasible for delivering child maltreatment programs typically delivered in the home (40, 41). Emerging evidence suggests that virtual and/or technology-augmented delivery of evidence-based child maltreatment programs originally designed to be delivered in person decrease program access barriers, improve program efficiency, increase family attendance and service completion, and lead to similar program outcomes as in-person delivery (41–44).

Yet, barriers exist for virtual delivery, including technology access, concerns about the quality of care, and how differing delivery approaches can impact program fidelity. Many questions remain, and thus, a comprehensive synopsis of technology use in practice across parent and child-level programs is needed to summarize evidence on not only these technological developments, but the effectiveness of these adaptations on measured outcomes among parent and child populations. This scoping review provides a contextualized overview and summary of the use technology in evidence-based parenting and child programs serving families impacted by child maltreatment, and the impact of technological methods, as available, on measured outcomes over the past two decades.

## Materials and methods

### Study design

A scoping review was chosen, in contrast to a systematic review, as the method of choice to synthesize and describe the breadth of available studies using various research methodologies without analytic comparisons, allowing for a comprehensive summary of the published literature that can guide future research directions as well as practice and policymaking (45). In accordance with Arksey and O'Malley's (46) framework, this scoping review included five stages of methodology: (1) development of specific review questions, including the definition of participants and establishment of inclusion criteria, (2) a comprehensive search of the published and grey literature; (3) selection of relevant studies; (4) charting of identified data; (5) summary and discussion of the results in the context of the field and future directions.

### Identification of objectives and research questions

The objective of this research was to understand how technology can be better leveraged and understood to improve evidence-based child maltreatment programs. Our goals were to describe the evidence-based programs, the nature of technology incorporated, the nature of change across measured maltreatment-related outcomes.

As such, we posed the following questions:

(1) How has technology been used in evidence-based parenting and child/youth programs for child maltreatment from 2000 to 2023?
(2) What are the effects of technology-enhanced programs measured population outcomes?

### Definitions

For the purposes of this review, evidence-based programs were defined as prevention programs at the parent level, or interventions at the child level rated as well-supported, supported, or promising through the Scientific Rating Scale established by the California Evidence-Based Clearinghouse (CEBC).

This review presents findings on two distinct populations affected by child maltreatment: parents and children. Parent populations were defined as the primary caregivers who are at-risk of perpetration or who have perpetrated child maltreatment. Child populations were defined as child or youth victims of child maltreatment, under 18 years of age.

### Inclusion criteria

Eligibility criteria for studies were established using the PICO framework (i.e., participants, interventions, comparators, outcomes) as seen in Table 1. In general, this review included studies published in English from 2000 to 2023 on evidence-based programs for child maltreatment prevention among at-risk parents or evidence-based treatment for sequelae among children 0–18 years of age exposed to abuse. Of note, the year 2000 was chosen as a key period when close to 50% of Americans were using the internet (45), or likely used a cell phone (47), or owned a computer (48).

**Table 1:** PICO overview.

Population of focus	At-risk parents for child maltreatment perpetration or youth 0–18 years of age experiencing child maltreatment Not limited by geography, ethnicity, or race
Intervention	Evidence-based programs as identified by the CEBC to prevent child maltreatment risk among caregivers or address child maltreatment sequelae among children Must report on a technological adaptation or modification for inclusion
Comparator	This scoping review will be inclusive of all comparators, which may include, care as usual, the original evidence-based program as prescribed, or no comparators
Outcome	Behavioral, mental health or well-being indicators for target populations

In addition, studies must have tested technological supports in the intervention for inclusion. Studies with provider informants reporting on population outcomes were also included in this work. All study designs were eligible for inclusion, such as pilot studies, qualitative accounts, cross-sectional studies, and effectiveness/efficacy trials. Exclusion criteria included protocol papers, discussion papers and field notes that did not include empirical data.

### Identification of relevant published literature

As mentioned, the review was designed to be inclusive of published peer-reviewed articles and select grey literature (e.g., conference abstracts, dissertations, reports) in the past two decades, between 2000 and 2023. The search extended across several subject areas including public health, psychology, medicine, education, and social work. Publications were identified using the following databases: Embase, Education Source, Academic Search Complete, PubMed, PsycINFO, CINAHL, and Psychology and Behavioral Sciences Collection. As recommended by Arksey and O'Malley (46), we worked with university librarians with expertise in scoping reviews to identify key words and execute a comprehensive search across databases. Searches were performed using identified key words and related MeSH terms across five domains of interest (see Tables 2, 3 for list of keywords and search strings, respectively).

**Table 2:** Conceptual domains of interest and related keyword terms.

Main concepts	Keywords
Child maltreatment	Child maltreatment, child abuse, child trauma, neglect, child physical abuse, child sexual abuse, child neglect
Evidence-based programs	Intervention, behavioral intervention, treatment, program, therapy, evidence-based program
Technology	Mhealth, digital, smartphone, app, iPhone, mobile health, e-health, android, technology, telehealth, tablet, online, virtual delivery, internet, virtual
Parent/child	Parents, or caregivers, guardians, mothers, fathers, foster parents
Child	Youth, children, child, adolescent, foster child

**Table 3:** Database key word search strings.

Database	Key Word Strings^a^
Academic Search Complete CINAHL Education Source Embase PsycInfo Psychology and Behavioral Sciences Collection PubMed	“Guardians” or “Parents” or “caregivers” or “mothers” or “fathers” or “youth” or “children” or “child” or “adolescent” OR “parents” [MeSH]) AND (“Child maltreatment” or “child abuse” or “child trauma” or “neglect” or “child physical abuse” or “child sexual abuse” or “child neglect” or “child maltreatment” [MeSH] or “child sexual abuse” [MeSH] or “battered child syndrome” [MeSH]) AND (“Mhealth” or “digital” or “smartphone” or “app” or “iphone” or “mobile health” or “ehealth” or “android” or “technology” or “telehealth” or “tablet” or “online” or “virtual delivery” or “internet” or “virtual” or “digital technology” [MeSH] or “internet-based intervention” [MeSH]) AND (“Intervention” or “behavioral intervention” or “treatment” or “program” or “therapy” or “evidence-based program” or “behavior therapy” [MeSH]) Language—English, Date: Jan 2000–Dec 2023; Population Group: Human

^a^ MeSH Terms were used as applicable to databases.

### Selection of relevant studies

A preliminary search of PubMed was conducted using key word string combinations and established MeSH terms. This step was taken to confirm the relevance of key terms in netting eligible articles. A comprehensive search across listed databases was conducted following the completion of the pilot literature search. Additional steps were taken to screen reference lists of retrieved and relevant publications. Special issues from leading family violence journals were also reviewed for completeness of search.

The research team uploaded retrieved citations into Endnote (49), a reference and citation manager, to facilitate organization of studies and duplicates removal. Following duplicates removal, the primary researcher created a comprehensive library that was used to screen relevant caregiver- and child-level publications. Screening procedures were conducted as follows: Three members of the research team (AT, MR, JG) independently screened titles and abstracts for eligibility criteria, keywords, and MeSH terms. All noted programs in publications were cross-compared to the CEBC for evidence-based intervention research ratings. Full-text articles were retrieved for all potentially relevant studies. We also retrieved full texts of articles with abstracts with ambiguous relevance during screening. At this stage, the three researchers screened retrieved articles for inclusion in results. Discrepancies were resolved by the lead researcher (AT) as they arose.

### Data charting

Identical charting procedures were made for caregiver and child population findings as follows: Upon establishment of final eligible articles, two researchers used Microsoft Excel to first extrapolate study characteristics, including but not limited to: author information, publication year, publication type, population and sample size, location, intervention and evidence-based rating, technological adaptation identified, study design, and outcomes and/or key findings. Team members met as needed to discuss iterative changes as they emerged.

## Results

**Figure 1:**
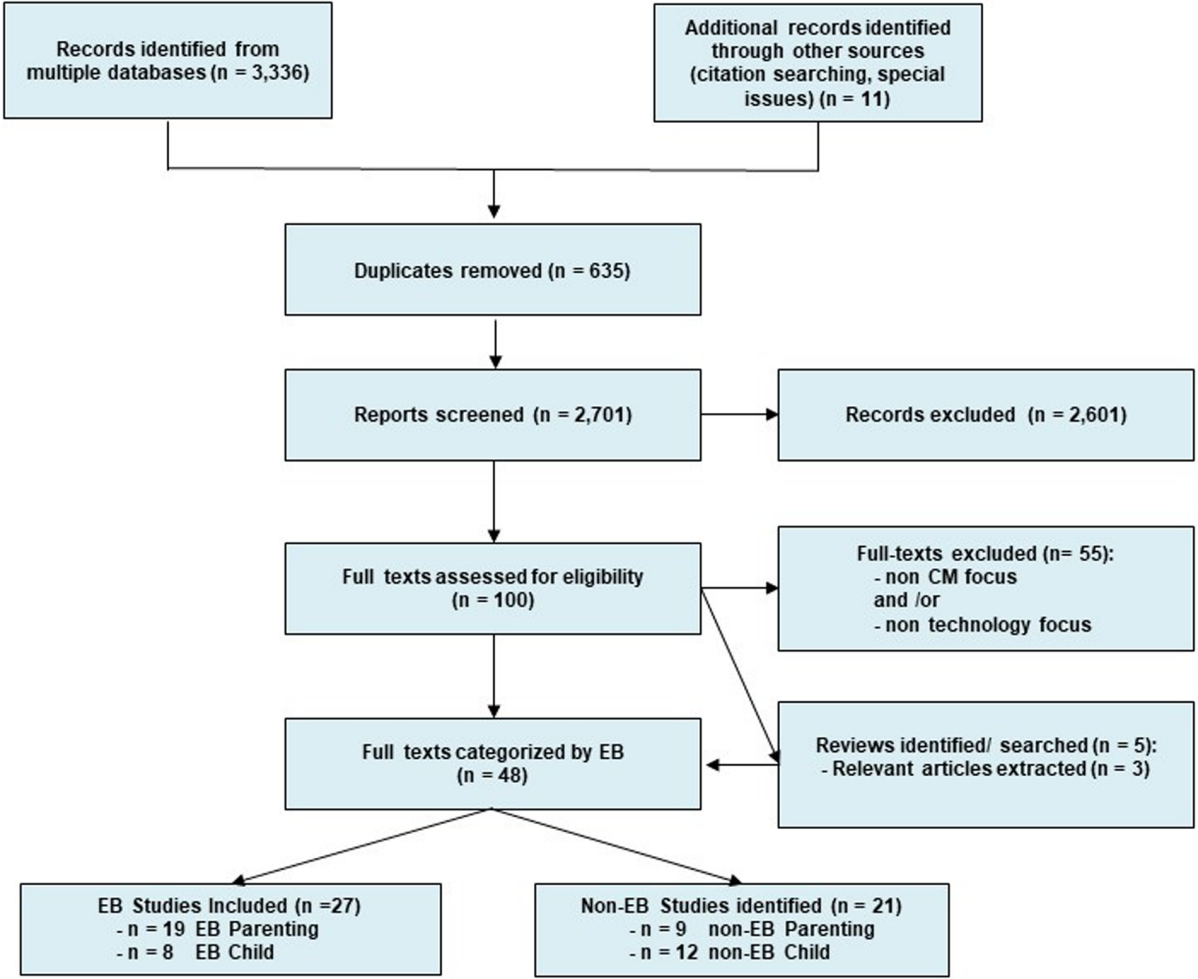
Scoping review PRISMA flowchart.

Figure 1 illustrates the flowchart of selection of final articles in the study. Using the previously mentioned key words, an initial search yielded 3,336 articles. Upon removal of 635 duplicate articles and an initial screening process removing 2,701 titles, we assessed 100 full text articles for eligibility criteria. Our final set of studies included 25 peer reviewed articles, and 2 peer-reviewed abstracts examining evidence-based parenting (*n* = 18 peer-reviewed articles; *n* = 1 peer-reviewed abstract), or child-level programs (*n* = 7 peer reviewed articles; *n* = 1 peer-reviewed abstract) using technology. Two major uses of technology across all programs emerged and were coded during data extraction—(1) remote programmatic delivery, or (2) programmatic enhancement. We define remote programmatic delivery as the complete, or hybrid use of virtual technology such as videos, recordings, or video conferencing platforms via data or internet channels to synchronously or asynchronously deliver all program components. Programmatic enhancements were defined as the use of technology to augment therapeutic content and goals in-person or between sessions. Below we summarize findings across the parent and child-level programs and report on the use or technology, and evaluation of program outcomes as available. An overview of all evidence-based parenting and youth/child programs identified can be found in Table 4.

**Table 4:** Overview of evidence-based parenting and child programs.

Evidence-based program	Program targets	Program as prescribed	Application
Parenting Programs			
ACT-Raising Safe Kids (50)	The ACT Raising Safe kids is a parenting program that teaches positive parenting and parent anger management skills. The program also creates a community support system with other parents in the program.	Eight or nine weekly 2-hour sessions that include didactic instruction, group activities, discussion, and role play.	Lotto et al. (51)
e-PALS Baby-Net Program (52)	e-PALS is an online version of Play and Learning Strategies (PALS) that uses online content to strengthen the parent-child relationship by teaching parents how to interpret and respond to babies’ signals and utilize rich language.	Eleven 90 min home visits which include video of parent and child interacting and video playback to review behaviors.	Baggett et al. (53) Feil et al. (54)
Incredible Years (55)	The Incredible Years program strengthens parenting and fosters parental involvement in child education to improve children's skills and conduct.	Two to three-hour parent group sessions are provided once per week for 12–20 weeks.	Camero et al. (56)
Healthy Families America (57)	Healthy Families America is a home-visiting program aimed at promoting positive parent-child interactions and secure attachments and building protective factors.	Home visits occur for 50–60 min weekly. After standardizing family progress, visits are conducted on a bi-weekly, monthly, then quarterly basis until graduation.	Ondersma et al. (58)
Parents as Teachers (PAT) (59)	The PAT program is a home visiting program used to increase parent knowledge about child development and parenting practices, identification of child developmental delays and health issues, prevent child maltreatment, and boost children's school readiness and success.	Two-year (recommended) program including home visits, group meetings, and annual child health screenings. The number of one-hour home visits ranges from 12 to 24 home visits depending on needs.	Traube et al. (60)
SafeCare (61)	The parent-child and/or parent-infant interactions module teaches how to interact positively with their child, perform engaging activities, and address their children's challenging behaviors.	SafeCare delivers three modules (Health, Safety, Parent Child/Infant Interactions) by trained professionals within the home. Each session typically lasts an hour. Modules can be delivered independently or in any order. The entire program can be delivered in 18 sessions.	Bigelow et al. (62) Gaskin et al. (63) Guasteferro et al. (64) Lefever et al. (26) Jabaley (65])
Tele-ABC: virtual Attachment and Biobehavioral Catch-up (ABC) (66)	The ABC program has 3 components: teaching caregivers to interpret children's behavioral signals and provide nurturing care, helping caregivers provide a responsive and positive environment to enhance children's’ behavioral and regulatory capabilities, and helping caregivers reduce behaviors overwhelming or frightening to a child.	Ten, weekly, one-hour sessions that incorporate video-feedback and homework activities.	Schein et al. (67)
Triple P (68)	The Triple P Positive Parenting Program is a five-tiered system that promotes parental confidence and competence, positive parenting strategies, child self-regulation, and parent communication.	The Triple P system consists of five tiers of intervention that vary in intensity. • Level 1: media campaign • Level 2: seminars and single-session meetings • Level 3: 1–4 sessions • Level 4: 8–10 sessions • Level 5: provides further support	Calam (69) Sanders et al. (70) Sanders et al. (71)
Triple P Online (72)	Triple P Online is an extension of Triple P that provides online support to parents to address challenging child behaviors, teach positive parenting skills, and promote emotional self-regulation.	Eight online modules are delivery virtually as well as video demonstrations and computer-assisted goal setting.	Sanders et al. (73)
Child/Youth Trauma Program			
Trauma-Focused Cognitive Behavioral Therapy (TF-CBT) (31)	TF-CBT is a joint child-parent psychotherapy model for children who have experienced a traumatic event and have significant emotional and behavioral difficulties.	Twelve to fifteen 30–45 min weekly sessions for the child and parent, separately. Near the end of the program, 30–45 min joint sessions are used.	Stewart et al. (39) Stewart et al. (74) Stewart et al. (75) Zinny (76) Orengo-Aguayo et al. (77)

### Evidence-based parenting programs overview

Nineteen studies (See Table 5) described or evaluated use of technology using data across the following eight evidence-based parenting programs: ACT Raising Safe Kids (*n* = 1), Attachment and Biobehavioral Catch-up (*n* = 1), Healthy Families America (*n* = 1), The Incredible Years (*n* = 1), Parents as Teachers (*n* = 1), Play and Learning Strategies (PALS) (*n* = 2), SafeCare (*n* = 7), and the Positive Parenting Program (Triple P) (*n* = 5). Data were available from five countries, including, the U.S., Australia, Brazil, Canada, and New Zealand, through single site and one multisite study. One study specifically focused on adolescent pregnant girls (54). Three studies provide reports in the context of the COVID-19 pandemic (41, 51, 56). Of these, two studies were conducted among providers engaged in parenting program delivery, who shared qualitative accounts of participant progress through the program (41, 42). A detailed description of findings across all studies is provided below, presented by type of technology used in programs.

**Table 5:** Use and effects of technology in evidence-based parenting programs.

Author	Name of adapted program and original EBP	COVID-19 context	Sample Size Location	Remote Programmatic Delivery	Programmatic Enhancement	Effect of Technology on Measured Outcomes	Participant Satisfaction/Engagement	California Evidence-Based Clearinghouse (CEBC)
Remote Delivery								
1. Baggett et al. (53)	e-PALS Baby-Net Program Adaptation of Play and Learning Strategies (PALS) program)	No	159 English and Spanish speaking, low-income mothers U.S.	Internet-based intervention with self-directed learning via videos; video recorded practice; remote coaching via calls Provided computers for participant use		Session dosage was associated with positive parenting and reduced potential for child abuse among high-risk group.	Most of the mothers at high risk for child maltreatment engaged in the intervention. Higher and lower risk groups had high completion rates (91% vs. 94%).	Yes (PALS); Child Welfare Relevance = Medium
2. Camero et al. (56)^a^	The Incredible Years Parents and Babies	Yes	36 English/Spanish Speaking mothers U.S.	8-week virtual workshop through a secured platform		Pilot is currently underway to evaluate parenting, attachment, and infant health outcomes	Interim findings among one group of 9 parents: Participants found follow-up communication to be beneficial, particularly for those who felt alone during the pandemic. Parents appreciated the opportunity to engage with other moms and professionals and learning ways to care for their children.	Yes (The Incredible Years); Child Welfare Relevance = Medium
3. Feil et al. (54)	Infant-Net Program e-PALS Adaptation of Play and Learning Strategies (PALS)	No	3 pregnant adolescent girls, 15–17 years of age U.S.	Interactive internet-based sessions, participant self-video recordings QuickTime multimedia presentations; video transfer for skill assessment Provided laptop with webcam for participants	Electronic bulletin board for peer communication	Preliminary findings indicate increases in knowledge about infant signals and responsive parenting behaviors.	High-very high satisfaction and usability ratings observed.	Yes (PALS-3); Child Welfare Relevance = Medium
4. Lotto et al. (51)	ACT-Raising Safe Kids	Yes	20 maternal-child dyads Brazil	Google Meet and Google Forms platforms used for full online delivery of group sessions, activities, homework		Significant increase pre- to posttest in emotional/behavioral regulation and sense of competence and decreases in coercive practices.	The online delivery version received greater acceptance among participants.	Yes; Child Welfare Relevance = Medium
5. Sanders et al. (71)	Triple P + “Families” (Triple P adaptation)	No	56 mothers	Videotaped “infotainment” delivery of Triple P, i.e., “Families”		Compared to waitlist control group, the Triple P TV condition reported decreased child disruptive problems and higher perceived parenting competence post-intervention and at 6 months follow-up.	86% of mothers watched all 12 videotapes. Consumer acceptability was high in the television condition.	Yes; Child Welfare Relevance = Medium
6. Sanders et al. (73)	Triple P Positive Parenting Program Online (TPOL) (Triple P adaptation)	No	193 mothers and fathers New Zealand	Internet-based session delivery, video demonstrations, computer assistant exercises		While effective in improving parenting practices, familial interactions, and problematic child behaviors, there were no differences between TPOL and the Triple P self-help workbook.		Yes; Child Welfare Relevance = Medium
7. Schein et al. (67)	Tele-ABC- virtual Attachment and Biobehavioral Catch-up (ABC)	Yes	70 parents U.S.	Full or hybrid telehealth delivery Provided devices or hotspots as available by agencies		Families in both telehealth and hybrid groups showed significant improvements in parental sensitivity and parenting behaviors including following lead and intrusiveness.		Yes; Child Welfare Relevance = high
8. Self-Brown et al. (41)	SafeCare	Yes	303 SafeCare providers across high-risk service agencies US. (n = 286); Australia, and Canada (n = 17),	Virtual delivery via Zoom, FaceTime, and Google Duo		Providers qualitatively reported positive but slower progression towards target skill goals among families in virtual delivery. No significant associations between SafeCare experience or country of residence with family engagement, or improvement.		Yes; Child Welfare Relevance = high
9. Self-Brown et al. (42)	Technology-Assisted SafeCare Takes Care (SC-TA) Adaptation of SafeCare	No	31 SafeCare providers across high-risk service agencies U.S.	Tablet-delivered, web-based program that provides video-based psychoeducation and modeling		n/a	Providers report general positive acceptance of technology among families, including advantages of video presentations, audio, and visual aids. Few families opposed to technology were less receptive to videos.	Yes; Child Welfare Relevance = high
10. Traube et al. (60)	Parents as Teachers (PAT)	No	84 English/Spanish parent-child dyads U.S.	HIPAA compliant, interactive video conferencing technology for virtual delivery. Provided tablets for families in need		n/a	Parents noted high user satisfaction with the program and educator, with > 89% noting equal or greater experiences compared to personal experiences with other in-person sessions.	Yes; Child Welfare Relevance = medium
Programmatic enhancement								
11. Bigelow et al. (62)	Cellular Phone- Enhanced Planned Activities Training (PAT) (CPAT) SafeCare Component	No	19 mothers U.S.		Provided cell phones to mothers for coaches to call between sessions, and supportive texts via phone or computer software: NotePager Pro 3.0 used by providers to text participants		Parents who received CPAT generally reported high satisfaction with content and their engagement with coaches via phone. Interim findings show those who received CPAT rather than PAT are less likely to miss appointments. Attrition rate was twice as high among PAT conditions compared to CPAT condition.	SafeCare- Yes; Child Welfare Relevance = high
12. Calam et al. (78)	The sex-episode “Driving Mum and Dad Mad” television series among families with disruptive children receiving group Triple P	No	723 biological mothers, fathers, or caregivers i.e. (stepparents, adoptive parents, foster parents and grandparents) Australia		Technology-enhanced viewing with a self-help workbook and extra web support (downloadable parenting tip sheets and audio and video streaming of positive parenting messages)	Parents assigned to the standard conditions were more likely to stay engaged and continue to the final stage of the program. Results indicated that there were significant improvements in dysfunctional parenting, parental anger, parental mood, and parental self-efficacy.		Yes (Triple P); Child Welfare Relevance = Medium
13. Gaskin et al. (63)	Parent-Infant Interactions (PII) of SafeCare	No	Single case; mother with an intellectual disability U.S.		Provided digital picture frame to participants in PII to capture photos of skill modeling and posed interactions	Preliminary findings indicate a strong increase in physical and non-physical parent-infant interaction skills post-intervention and at 1, 2, 4 months follow-up.	Parent expressed satisfaction with learning through frames and but discomfort with taking self- photos.	Yes; Child Welfare Relevance = high
14. Guastaferro et al. (64)	Parent-Infant Interactions (PII) module of SafeCare	No	4 maternal-infant dyads U.S.		Provided digital picture frame to participants in PII to capture photos of skill modeling and posed interactions	The intervention was associated with an increase in the demonstration of physical and non-physical skills.	General satisfaction with digital frame use	Yes; Child Welfare Relevance = high
15. Jabaley et al. (65)	Safety Module of SafeCare	No	3 mothers U.S.		Provided mobile device to enhance SafeCare via for in and between session communication via texts and calls between parent provider, and video data collection	Preliminary findings show iPhone intervention was associated with reductions in household hazards in all rooms. Texts were the most common communication format.	Parents reacted positively to the cell phone enhancement.	Yes; Child Welfare Relevance = high
16. Lefever et al. (26)	Parent Child Interactions (PCI) module of SafeCare	No	371 low-income maternal-child dyads U.S.		Provided mobile device to enhance in-between text and call communication between parent and provider. NotePager Pro 3.0 used by providers to text participants	Although no significant difference between PCI and cell-phone supported PCI (PCI-C), greater improvements in parenting behaviors and depression symptoms observed across both groups compared to waitlist control group one-year post intervention. Children in the PCI-C group were more cooperative and less aggressive than children who were in the waitlist control group. Lower rates of attrition among PCI-C compared to PCI group.		Yes; Child Welfare Relevance = high
17. Love et al. (79)	Positive Parenting Program (Triple P) Online Community (social media Triple P Online adaptation)	No	155 high-risk mothers and fathers U.S.		Use of a social network online community (including discussion boards, and postings), gaming features (e.g., avatars; badges to incentivize skills practice), and access via smartphones, tablets, computers	Significant improvements were seen in parental stress, parenting practices (over reactivity, laxness), and problematic child behaviors. These effects were maintained at six-months post intervention.	Parents valued the flexibility, anonymity, and shared learning within the intervention.	Yes; Child Welfare Relevance = Medium
18. Sanders et al. (70)	Triple P	No	3,000 English-speaking parents Australia		Local media and communication strategies such as television and radio broadcasts. Community access to telephone counselling support services Optional telephone consultation in group Triple P	Population trial results indicate increased awareness of Triple P among communities exposed to resources; significant reductions across coercive parenting, depression, and stress; no differences noted in support of confidence. Significant reductions in child-level clinical emotional symptoms in Triple P group compared to care-as-usual group. Significant reductions in child-level behavioral and emotional problems compared to care-as-usual group.		Yes; Child Welfare Relevance = Medium
19. Ondersma et al. (58)	Healthy Families America	No	413 At-risk, English-speaking women U.S.		e-Parenting Program: computer delivered modules augmenting Healthy Families sessions	E-Parenting Program group showed no significant improvement in depression and self-reported drug use from baseline to 6 month follow up and 6 month-12 months, compared to services as usual and control group	Parents were likely to recommend the program. e-Parenting Program received positive feedback following program completion. Participants providing high ratings across scored helpfulness, and respectfulness, and working alliance with providers. Average scores were noted for relevance.	Yes; Child Welfare Relevance = Medium

^a^ Peer-Reviewed Abstract.

#### Remote programmatic delivery

More than half of the included studies on evidence-based parenting programs (*n* = 10) reported on telehealth or online delivery of programmatic content. The majority of articles described full telehealth delivery of content. Only two described the use of hybrid, or asynchronous programmatic delivery to participants. Four studies noted provision of loaned equipment to facilitate remote delivery procedures to families (53, 54, 60, 67).

Measured improvements among participants following full telehealth program participation were noted across child maltreatment risk (51, 53, 65, 73), parenting skills and behaviors (51, 52, 63, 64, 67, 70, 71, 73), mental health (26, 60, 70), engagement in services (53, 62, 78) as well as child-level behaviors (26, 70, 71, 73, 79). Of note, two articles describe provider-level data, via qualitative inquiry, on parent acceptance or progression through remote delivery services. Self-Brown et al. (42) describe an early account of a web based SafeCare via tablet, in which providers noted positive reception among families, citing feasibility benefits to using audio and visual aids for programmatic delivery. Five years later, Self-Brown and colleagues (41) furthered virtual delivery work during the pandemic, citing virtual SafeCare delivery via web-based platforms among providers. While improvements across parental outcomes were noted, providers highlighted a slower progression towards targeted goals during virtual delivery compared to in-person sessions.

Among articles describing hybrid approaches and/or asynchronous delivery of services, Schein et al. (67) examined effects of telehealth as well as hybrid delivery of Attachment and Biobehavioral Catch-Up on parenting outcomes within the COVID-19 context, and cited significant improvements in parenting sensitivity and behaviors across both delivery formats. In a Triple P implementation study, Sanders et al. (70) cited the use of videotapes to facilitate content delivery, noting improvements across parenting confidence as well as child behavioral problems.

#### Enhancements

Eight studies on evidence-based parenting programs described alternative uses of technology, specifically to augment program delivery to families. Examples of technological enhancements included digital picture frames and mobile devices to facilitate in-person sessions, or direct external e-communication between parents and providers (26, 62, 64, 65), as well as web-supported audio and video content to supplement evidence-based program content (78). Of note, in many of these applications, participants were provided with needed technology devices during the intervention (26, 64, 65).

One additional technological enhancement was the optimization of the Healthy Families home visiting program though an internet-based, tablet delivered, eight session e-parenting program designed to address child maltreatment risk (58). The e-parenting program curriculum was grounded in three effective evidence-based models (i.e., SafeCare, motivational interviewing and cognitive retraining). Ondersma et al. (58) note the use of video-based skill modeling and feedback as part of the e-program. The enhancement demonstrated feasibility in its implementation, as well as positive reception among parents, but parent ratings of relevance were low. Improvements in maternal depression symptomology and perpetration risk were observed following intervention completion but were not maintained at 6 or 12 month follow up in comparison to services as usual and control groups (58).

Two studies also describe the use of online discussion forums as strategies for evidence-based programs to build group-based support systems among participating families (52, 79). One population-based study also described strategies for broad-based dissemination of programmatic material to increase program awareness in target communities (70).

### Evidence-based child focused trauma programs overview

Eight child-level intervention studies were identified for inclusion in this study (See Table 6), all examining effects of technology across one intervention, Trauma-Focused Cognitive Behavioral Therapy (TF-CBT). Data were collected from the U.S. (*n* = 6), Canada (*n* = 1), and Puerto Rico (*n* = 1). Importantly, only one study examined data collected during the pandemic COVID-19 context.

**Table 6:** Use and effects of technology in evidence-based child/youth programs.

Author	Name of EBP	COVID-19 context	Sample size Location	Remote Programmatic Delivery	Safety Plan	Programmatic Enhancement	Effect of Technology on Measured Outcomes	Participant Satisfaction	California Evidence-Based Clearinghouse (CEBC) Rating
Telehealth									
1. Stewart, Orengo-Aguayo, Cohen, et al. (39)	Trauma-Focused Cognitive Behavioral Therapy (TF-CBT)	No	*N* = 15 trauma exposed youth children 7–16 years of age U.S.	HIPAA-compliant videoconferencing software “Vidyo in school and home settings Adaptations during telehealth: digital books, Microsoft Office, chat features, digital games, video clips iPads provided to families for treatment.	+		Significant reductions in child-and parent reported PTSD, depression, and anxiety symptoms, and parent-reported externalizing symptoms, and total problem behaviors 0% attrition rated noted	Caregivers noted 100% satisfied with tele-health services in and convenience, rapport. The majority noted ease of use (86%). All parents recommended telehealth services	Yes; Child Welfare Relevance = high
2. Stewart, Orengo-Aguayo, Gilmore, et al. (74)	TF-CBT	No	N = 4 trauma exposed Hispanic youth 10–16 years of age U.S.	HIPAA-compliant videoconferencing software “Vidyo” Adaptations during telehealth: digital books, narrative tools, Microsoft Office, chat features, digital games, video clips iPads provided to families for treatment;			Clinical reductions across PTSD, and internalizing symptomology including depression and anxiety among all participants	All caregivers of youth all noted benefits of telehealth in reducing traveling time as well as increasing convenience	Yes; Child Welfare Relevance = high
3. Stewart et al. (75)	TF-CBT	No	3 African American youth 7–15 years of age U.S.	HIPAA-compliant videoconferencing software “Vidyo” in school setting. Editable Microsoft Office, and electronic documents during telehealth; video clips iPads provided to families for treatment;			Each of the participants had a reduction in self-reported PTSD and depression symptoms. Two participants no longer met PTSD diagnosis criteria after treatment.	All participants had positive reception of telehealth. Two participants note time-saving benefits via digital access. One participant reported comfort with clinician and using software during process	Yes; Child Welfare Relevance = high
5. Orengo-Aguayo et al. (77)	TF-CBT	Yes	N = 36 Puerto Rican trauma exposed youth Puerto Rico	Telehealth^b^	+		Significant reductions across child and caregiver self-reported anxiety, PTSD, and depression symptomology.		Yes; Child Welfare Relevance = high
4. Zinny (76)^a^	TF-CBT	No	*N* = 52 African American and Latino youth U.S.	Hybrid delivery—telehealth and in-person sessions			Youth who participated in TF-CBT plus case management and peer services had a significant improvement from pre- to post-test in PTSD symptoms. Differences between TF-CBT, TF-CBT hybrid vs. full telehealth were not examined		Yes; Child Welfare Relevance = high
Telehealth + programmatic enhancement									
6. Stewart et al. (80)	TF-CBT	Yes^c^	68 trauma-exposed, English or Spanish speaking children 7–18 years of age U.S.	Remote delivery in school or home HIPAA-compliant videoconferencing software “Vidyo” Electronic documents, video games Provided iPad as needed		Reminder phone calls, text messages	Significant decreases in child and caregiver reported PTSD		Yes; Child Welfare Relevance = high
Programmatic enhancement									
7. Caouette et al. (81)	TF-CBT augmented with Attachment Video-feedback Intervention (AVI)	No	33 children with sexual abuse disclosure ages 4–6 years Canada			AVI intervention: Videotapes clip of parent-child interactions used for parental feedback on observed parent-child relationship	Significant reductions in clinical child internalizing symptoms and self-reported dissociative symptoms following the intervention. No significant changes in clinical/self-reported externalizing symptoms or clinical dissociated symptoms Significant reductions in self-report but not clinical caregiver psychological distress or posttraumatic stress		TF-CBT: Yes; AVI: No Child Welfare Relevance = high
8. Davidson et al. (82)	TF-CBT	No	27 trauma -exposed youth and their caregivers U.S.			11 tablet-based TF-CBT activities during in-session treatment	Reductions across internalizing and externalizing symptoms, & PTSD were noted in both the tablet assisted (*n* = 18) and TF-CBT groups (*n* = 9).	Youth and caregivers both endorsed interactive tablet features, noting relevance and helpfulness in engagement during sessions. Youth in Tablet-assisted and TF-CBT groups both indicated mostly to very satisfied ratings for alliance with their providers	Yes; Child Welfare Relevance = high

a Conference abstract.

b Transition to telehealth within one month of the pandemic for ∼89% of active participants.

c Although data were not using a COVID-19 population, article was published in consideration of need for telehealth services.

#### Remote programmatic delivery

Most studies (*n* = 6) described implementation of synchronous, virtual delivery via HIPAA compliant telehealth video conferencing software such as “Vidyo” to delivery program content (39, 74–77, 80). In several instances, families were provided with iPads during therapy to facilitate telehealth delivery [for examples, see: (74, 75, 80)]. As part of the TF-CBT telehealth process, several studies also cited the use of technological adaptations during delivery to meet the remote needs of children and youth. Sessions may have included use of digitized materials such as books, games, and video clips to convey content to clients. Additional software, including Microsoft Word and Adobe, were mentioned as applications allowing participants to make edits to documents that were shared with providers (39, 74, 75, 77, 80). Of note, one study also described the use of both remote delivery and supplemental enhancement strategies to increase engagement between sessions (80). In this work, providers contacted participants via calls and text messages for session and homework reminders.

Although safety assurance plans for youth were likely implemented during the pandemic, only two telehealth-based studies explicitly alluded to inclusion during implementation. For example, Orengo-Aguayo et al. (77) describe the use of consultation calls to review safety concerns among therapists as treatment sessions were conducted, whereas, Stewart and colleagues (39) detail strategies in school and home settings including regular communications and presence of school staff and presence of caregivers or trusted contacts during sessions.

#### Technological enhancements

Two studies were identified that presented unique augmentations to TF-CBT. Both studies were also published using data collected prior to the pandemic. While varying in approaches and purpose, both studies noted significant improvements across internalizing symptomology and post-traumatic stress disorder among participating youth. First, Davidson et al. (82) piloted “tablet TF-CBT”, which included 11 tablet-based activities targeting constructs within the TF-CBT model and general mental health programs, designed to increase provider-client engagement. These activities were built in consultation with providers and considered embedded under the category of “available tools for providers” in the fidelity monitoring process. Second, a Canadian study by Caouette et al. (81) piloted the embedment of an Attachment Video-feedback Intervention (AVI) with TF-CBT, to measure changes in parent-child relationships. AVI was integrated as a video-feedback component for caregivers during psychoeducation to address parent-child interactions. The authors describe that this discussion added half hour to session length, and that protocol for each intervention was followed as prescribed (81).

## Discussion

This scoping review summarizes the use technology in evidence-based parenting child maltreatment prevention programs and child trauma therapy programs over the past two decades, inclusive of the recent COVID-19 pandemic years. We identified a total of eight parenting-focused programs, and one child-focused program across twenty-seven published works examining the use of technology in practice prior to, and since the onset of the pandemic. Our findings highlight the consistent presence of technology in remotely delivering services, and/or to enhance program content among target populations. Irrespective of the classification, technological advancements in practice were generally associated with positive parent and child mental health and behavioral outcomes in all geographic contexts and sample populations.

Although these results support the integration of technology in practice, the observed positive impact can be limited with low buy-in or feasibility in practice. However, review findings among parents and children across the 11 studies measuring participant satisfaction on the use of both telehealth and explored digital enhancements were generally encouraging. Indeed, these studies noted favorable reception or satisfaction rates among users, which may be a proxy indicator of perceived quality or engagement within these technologically advanced evidence-based programs. Conflicting findings, as with Ondersma et al. (58), may suggest otherwise, where positive reception to technology-based changes to programs did not equate to perceptions of relevance or positive program effects as observed. It is important to note; however, that the software included in this study, though very advanced at the time of the trial (2006–2009), holds very little relevance to the advancements that emerged in the last decade, and this could be a driver of the relatively poorer reception. Additionally, parent and child comfort and experience with using technology over the last decade has dramatically increased and has become standard in our social communications and general experiences with education, which could also substantially increase perceived relevance and satisfaction.

Other studies in this review, such as Baggett et al. (53) have shown that other feasibility indicators, such as increased session dosage, may be associated with stronger program outcomes. Collectively, these data emphasize that the investigation of associations between implementation measures and participant engagement, as well as with target outcomes among programs adopting technology is in its early stages at best, and even less explored in relation to specific uses such as telehealth and/or technological enhancements. Studies should include additional measured constructs across quality, dosage, and adherence for greater understanding of optimal approaches during program implementation.

While not a key focal population of this review, providers as the implementing agents of these evidence-based programs are a non-traditional but important group that can enhance our understanding of the feasibility and effectiveness of technological integration. Noting provider voice, in addition to clients, as part of development and testing of technology-based delivery approaches and augmentations is of utmost importance. Without provider or therapist buy-in to the use of technology as part of evidence-based practice delivery, there will be challenges with implementation. For example, Stewart et al. (74) described strong satisfaction amongst therapists with their telehealth delivery process, noting both comfort in using telehealth equipment as well as interactions with youth over digital technology. In contrast, in Self-Brown's (41) study on SafeCare implementation during the pandemic, providers described the logistic struggles faced by their families during telehealth delivery, as well as barriers in formatting program content and with conducting observational assessments using virtual delivery. General studies on therapist perspectives have also documented similar (83), as well as novel reflections on remote delivery considerations since the pandemic onset, such as quality and effectiveness of treatment (83–86), safety management in the home environment (85–87) and even provider virtual fatigue (83, 84). Such responses are key assessments that can shape directions for best practices for using technology in the context of human services.

### COVID-19 context

An interesting observation from this review was the limited number of articles focused on technology use and effectiveness in the context of parenting and child programs published since the onset of the pandemic. This may be due in part to the arduous and lengthy process of peer-reviewed publication, which can take up to 2 years from time of submission to publication. However, the aforementioned recommendations across agencies to adopt telehealth suggests that many evidence-based programs were, indeed, utilizing such technology to meet the needs of families during the pandemic. The limited available evidence is promising, but more studies are sorely needed in this area.

Moreover, it is critical to explore how well these programs approached, or are approaching, implementation-related outcomes, especially fidelity monitoring, or protocol adherence measurement, in real-world practice as pragmatic adaptations during delivery were likely to occur. Fidelity is critical as programs implemented as designed are noted to achieve positive outcomes (88–90). Several of the parenting and child program studies published prior to the pandemic describe formal fidelity monitoring for telehealth as well as technological enhancements [data not shown; for examples, see: (42, 60, 82)]. Evidence-based parenting programs such as Parents as Teachers established support systems for technical assistance during telehealth delivery (60). In contrast, in their web-based application of SafeCare, Self-Brown and colleagues (42) note use of a provider fidelity checklist that accommodated adaptations made during the implementation of their web-based program. However, in the COVID-19 context, few have documented evidence of strong provider fidelity during implementation of a telehealth program (67, 77, 91, 92), and only one, included in the current study, with likely related effectiveness in improving parental outcomes (67).

### Practice-based considerations

There is no dispute that real-time, digital delivery of programs can reduce geographic barriers for families who are likely burdened with numerous stressors. Our findings suggest that telehealth and programmatic enhancements are effective in increasing participation and improving outcomes among parent, youth, and child populations. Yet despite general satisfaction, positive indications of feasibility, and the unique position of telehealth options to alleviate geographic restrictions, developers and practitioners must continue to address formidable challenges around general program enrollment in addition to long waitlists as seen in child trauma programs*,* which may not be fixed with technology advents. Irrespective of how engaging a program may be, no observable effects will be noted without service-level supports and strong recruitment and retention strategies. Research must also focus on how well remotely delivered programs can effectively assess child safety, a hallmark of the child protection system in child maltreatment response. In other words, even in the presence of safety assurance plans, are providers able to accurately assess child safety and maltreatment risk, observing the home environment and key parent-child interactions?

Nonetheless, the use of various technological devices reflects the market of options available to assist with programmatic dissemination. As cell phones and tablets become more ubiquitous in use (47), these devices may become appropriate choices to engage with participants in and out of sessions and expand the reach of programmatic content. Uptake and incorporation of such technology in standard practice does come with caveats; as the question of digital equity regarding access to cellular data and connectivity remains (41), limiting the ecological validity of positive findings across highlighted efficacy studies in this review. We must continue to consider the present digital divide exacerbated by the pandemic (93), which faces many vulnerable families who are often the targets of these programs. One solution seen among studies was the provision of internet ready devices, which may assist with improving equity in access to care and higher engagement rates among parent and child populations. Yet, the removal of resource constraints among families will not address the logistic barriers seen at the provider level. For example, in the context of SafeCare during the pandemic, providers described logistic barriers with virtual translation of program content intended for in-person assessment, in which they experienced difficulties modeling skills and home assessments (41).

A key question regarding the inclusion of more technology-based applications in evidence-based programs then becomes, what information is potentially lost or enhanced in the process? In one school of thought, Mowbray et al. (94) classify essential programmatic content as structural (i.e., key components), or as process related (i.e., interactions, and rapport). If left unaddressed, logistic limitations could prohibit delivery of core program components and in turn, expected outcomes. It is encouraging to note that some programs are specifically addressing logistical limitations through tailoring of process-related content while maintaining structural content (i.e., fidelity to the model) when delivering interventions via telehealth (74). Promisingly, parent, youth and child accounts among included studies suggest that telehealth experiences and digital enhancements did not interfere with rapport building with providers (60). As we shift towards delivery with digital influences, programs should maintain some flexibility in delivery approaches, but rigor to the core components of the curriculum that is key to the mechanistic drivers of outcomes must be incorporated to ensure expected outcomes. Moving forward, technology could also be used in precision home visiting to strengthen the implementation of core components and efficiency of programs to serve the needs of diverse populations.

While our review captured the application of technology in evidence-based programs for child maltreatment, it is important to note additional existing literature in this area was not captured by through our search string or database selection. For example, artificial intelligence is a growing area of interest in data modeling of violence prevention to identify algorithms for perpetration and prevention (95). The current review did not include search parameters for this novel interface. However, with additional exploration, this technology will be important to assess within evidence-based programs in the future. Further, many established programs, with high relevance for preventing maltreatment and published technology research, were not captured because the technology-based modifications were applied to other target populations not captured in our search. For example, the Parent-Child Interaction Therapy (PCIT), an evidence-based program targeting child behavioral or externalizing mental health symptomology has been modified for remote delivery in its Internet delivered PCIT format, which uses comparable videoconferencing technology among families (96, 97). Though PCIT has also been shown to positive impact families at risk for child maltreatment (98) these studies focused on families with children experiencing significant behavioral issues (96). Several other non-evidence, based but emerging programs also describe digital advancements to promote outcomes, or are original digital interventions providing treatment (see Tables 7, 8). As one example, studies on smartphone app interventions, such as the JoyPop application which is based on trauma-informed principles, have demonstrated improvements in resilience and mental health symptomology and among young adult populations with a history of adverse childhood experiences. Preliminary evidence also supports the incorporation of JoyPop among both evidence-based parent (99, 100) and child programs (101). Such advancements represent new directions in technology providing rapid access to behavioral care.

**Table 7:** Non-evidence-based parenting interventions using technology.

Author	Name of EBP	How technology is used
1. Fogarty et al. (102)	Parenting Skill Development and Education (PSDE) Service	Six-week telehealth program to support parents and address child maltreatment risk
2. Gülırmak and Orak (103)	Web-based distance education	A six-week web-based distance education program to increase parental awareness of child abuse and appropriate attitudes toward child rearing
3. Lamberton et al. (104)	Netmums Parent Support Project	Netmums online site provides a forum for parents, evidence-informed advice, and support from staff, and connects parents with partner and local agencies.
4. McKenzie et al. (105)	Make Safe Happen® app	Mobile application with child safety information based on age and type of room, safety checklists, and links to home safety products.
5. Murray et al. (106)	Unnamed parent training program	Text message reminders
6. Oliveira et al. (107)	Video-feedback Intervention to Promote Positive Parenting and Sensitive Discipline (VIPP-SD)	Home-based intervention with video feedback in which caregiver/child interactions are recorded for analysis and discussion, and caregivers receive content based on feedback
7. Hodes et al. (108)	Video-feedback Intervention to Promote Positive Parenting and Sensitive Discipline (VIPP-SD)	Home-based intervention with video feedback in which caregiver/child interactions are recorded for analysis and discussion so the home visitor can help parents develop skills and reinforce sensitive parent behaviors.
8. Inouye et al. (109)	Wellness in the Home (WITH)	Plain old telephone service video technology was used to conduct video conference sessions at least once a week throughout participation in the WITH program.
9. van Leuven et al. (110)	All Children in Focus (ABC) program	Video conferencing software during the pandemic

**Table 8:** Non-evidence-based child/youth program using technology.

Author	Name of non-EBP	Use of technology
1. MacIsaac et al. (111)	JoyPop^TM^ mobile app	JoyPop^TM^ app has activities and resources to promote resilience and emotion regulation
2. McDonald (112)	Active Art therapy for Children in the Community	A digital health intervention to train art therapists
3. Calam et al. (69)	Computer-assisted interview	Computer program allows expression of experiences and emotions using various settings and people
4. Lange and Ruwaard (113)	Therapist-assisted web-based treatment	Therapy (including components of cognitive behavioral therapy and psychoeducation) and therapeutic assignments were completed through a web-browser
5. Constantino et al. (114)	MIVO	Email device was used to connect mothers and children who experienced abuse with a nurse
6. Kaier (115)	Tele-ERRT-C: tele videoconferencing Exposure, Relaxation, and Rescripting Therapy for children	Tele videoconferencing delivery of Exposure, Relaxation, and Rescripting Therapy for children
7. Moss et al. (116)	Un-named home visiting program to improve parental sensitivity and child attachment	Video-feedback was used to discuss parental feelings and behaviors and provide feedback on parental skills.
8. Endendijk et al. (117)	Vil Du? Video game	Video game that allows child to re-enact sexual abuse experiences using characters in a game
9. Mast et al. (118)	Internet-based Interacting Together Everyday: Recovery After Childhood TBI (InTERACT)	A web module consisting of readings and videos is watched by the caregiver and then the caregiver and therapist videoconference to role-play, watch interactions with child, and review module
10. van Rosmalen-Nooijens et al. (119)	Feel the ViBe (FtV)	Internet-based support intervention that includes informational pages, a forum, a chat, and a “connect to expert” feature that connect the user to a community manager.
11. Wagner et al. (120)	Internet-based imagery rescripting intervention	Internet-based writing assignments based on CBT and imagery rescripting components which were reviewed by psychiatrist who provided written feedback.
12. Castro et al. (121)	Social intelligence training	Web-based social intelligence training (SIT) with content displayed through audio- and visual methods

In consideration of the increased use of technology-based applications in evidence-based practice, we must also assess whether the cost of these advents counterbalances the magnitude of benefits, and sustainability of programs, or contributes to the limitations observed in practice. Only through reliable evaluation of these programmatic advancements can we weigh the cost-effectiveness in comparison to program efficacy. However, researchers and practitioners encounter limitations through federal funding requirements and current grant infrastructure, which prohibit rapid dissemination of quality science in emergent situations such as the pandemic. Concurrently, published findings then become irrelevant as reported upon technology become obsolete in the face of newer technological innovations entering the field.

## Conclusions

This scoping review is the first to provide a comprehensive examination of uses and effects programs through technology enhancements, as applicable, across evidence-based CM programs for both parent and child populations. The incorporation of technology presents exciting possibilities for program success for many models and the pandemic represents an era of novel directions for the field. However, the notably few available studies are an indication of the need for extensive exploration of the role of technology in practice. Only with such research can we definitively comment on its true utility. Further, the benefit of these programs is based on their effectiveness in achieving positive outcomes for parents, youth, and children. A prominent challenge of the field will be to identify effective means of implementing programs with technology in a sustainable manner. We have growing opportunities to explore innovative technologies in practice to meet the needs of families and must continue to explore methods to promote behavioral change through empirically validated research testing the rigor of technology-based strategies in practice.
